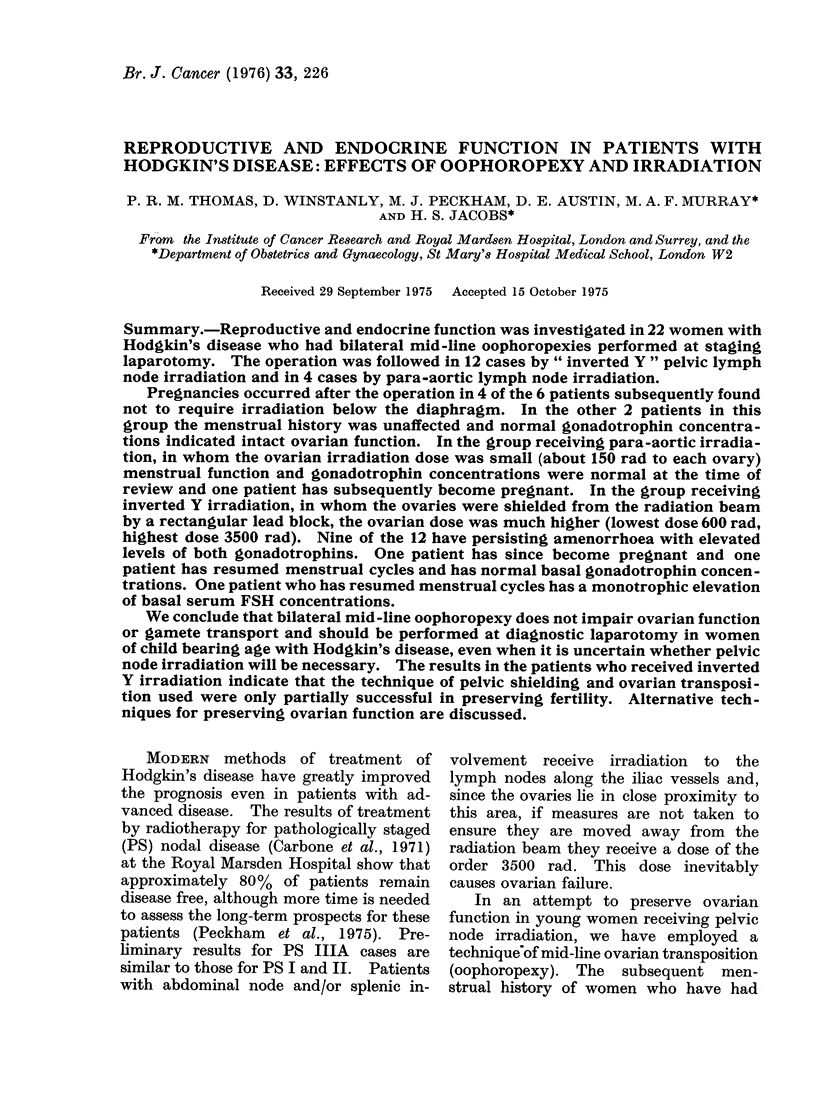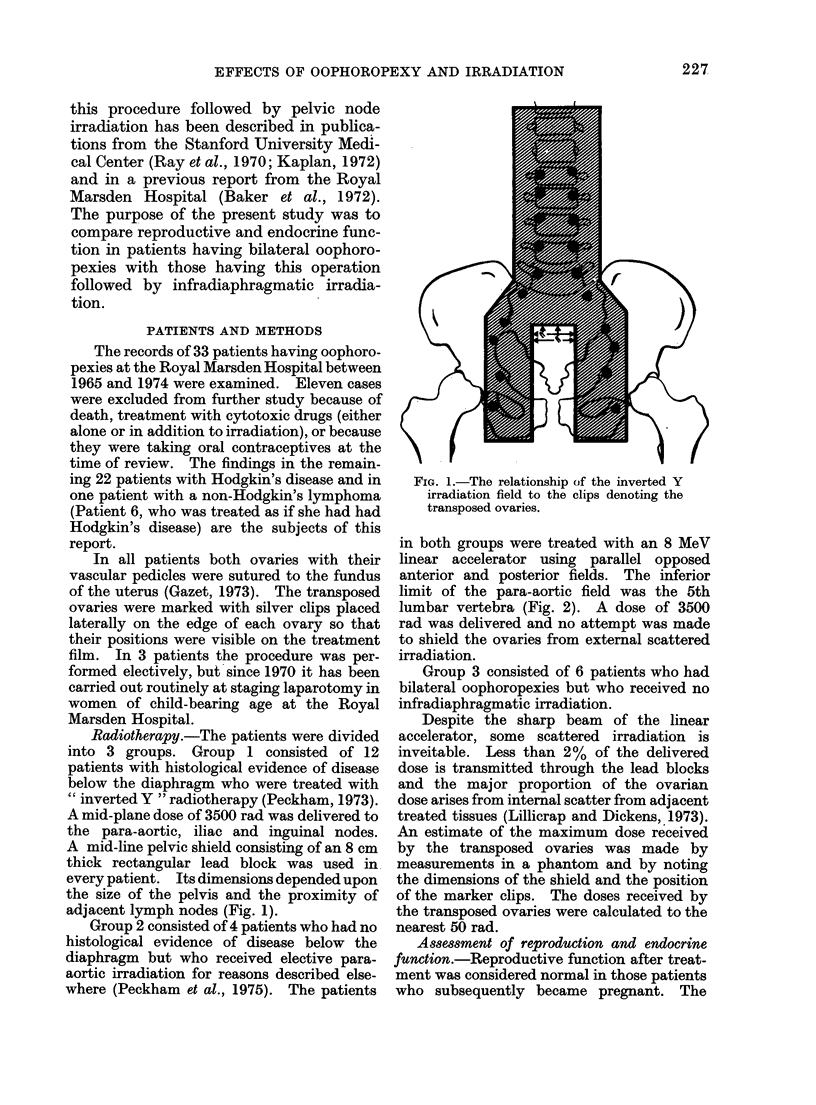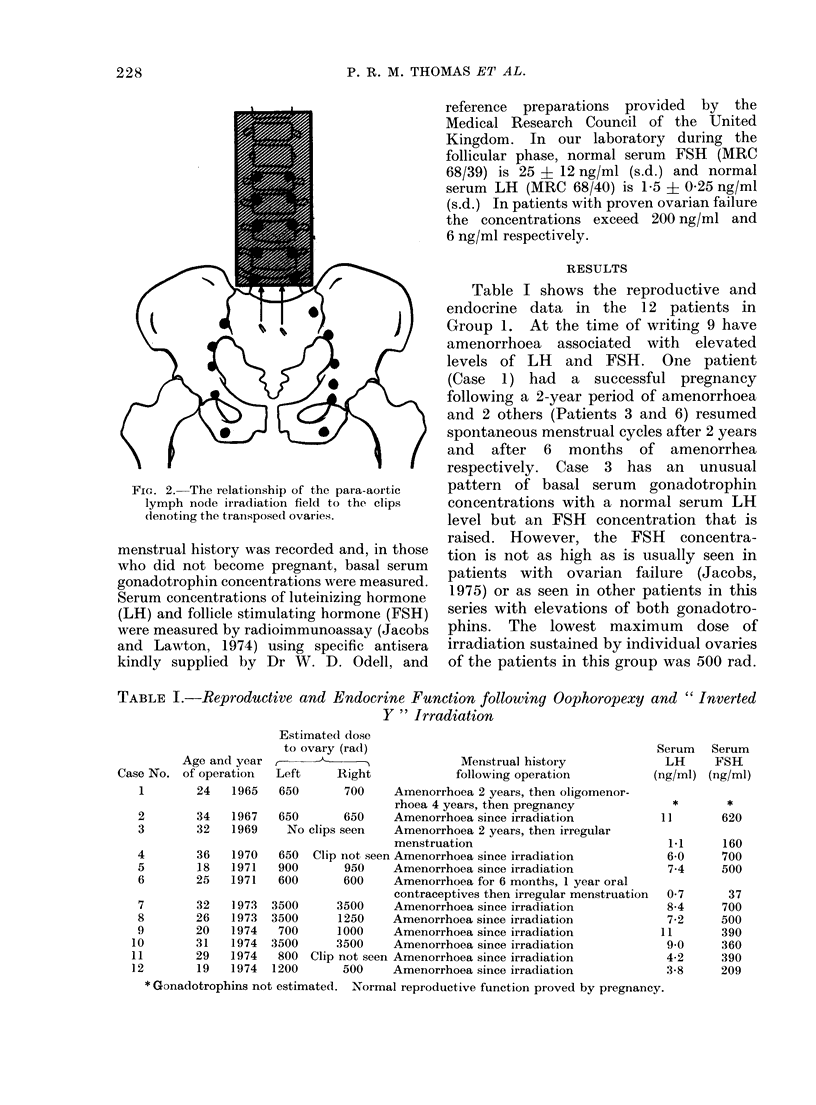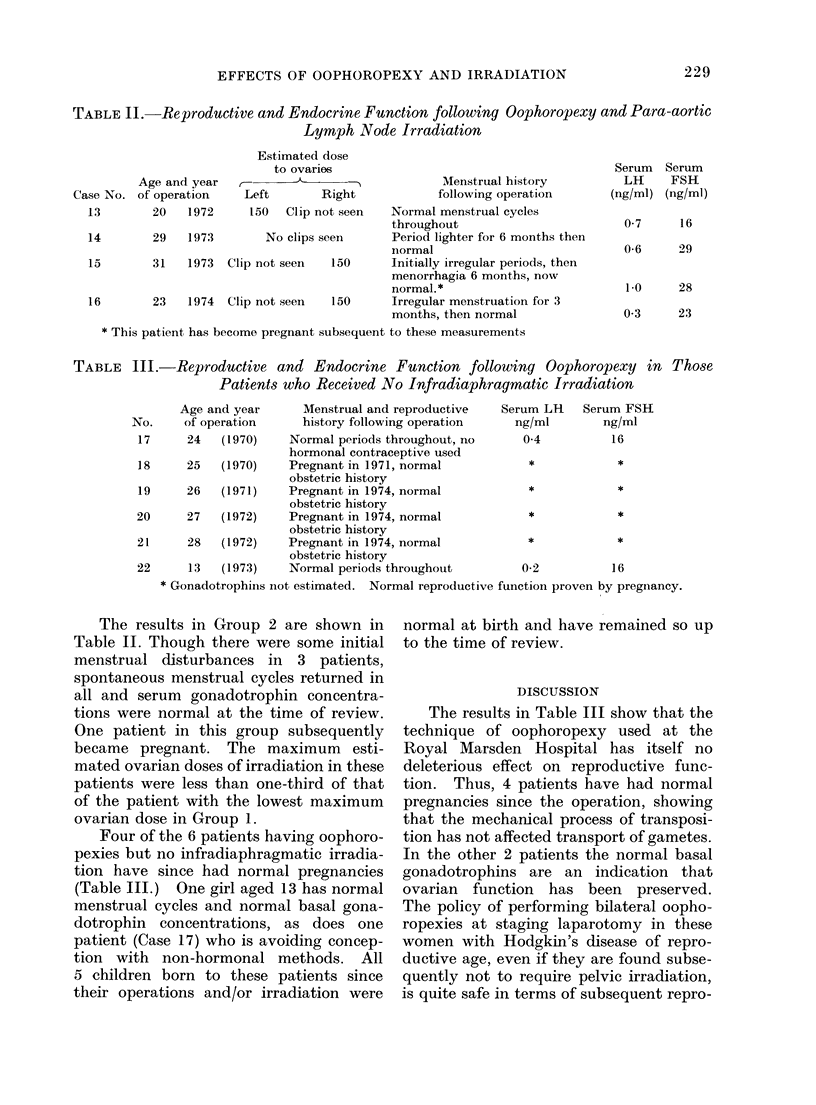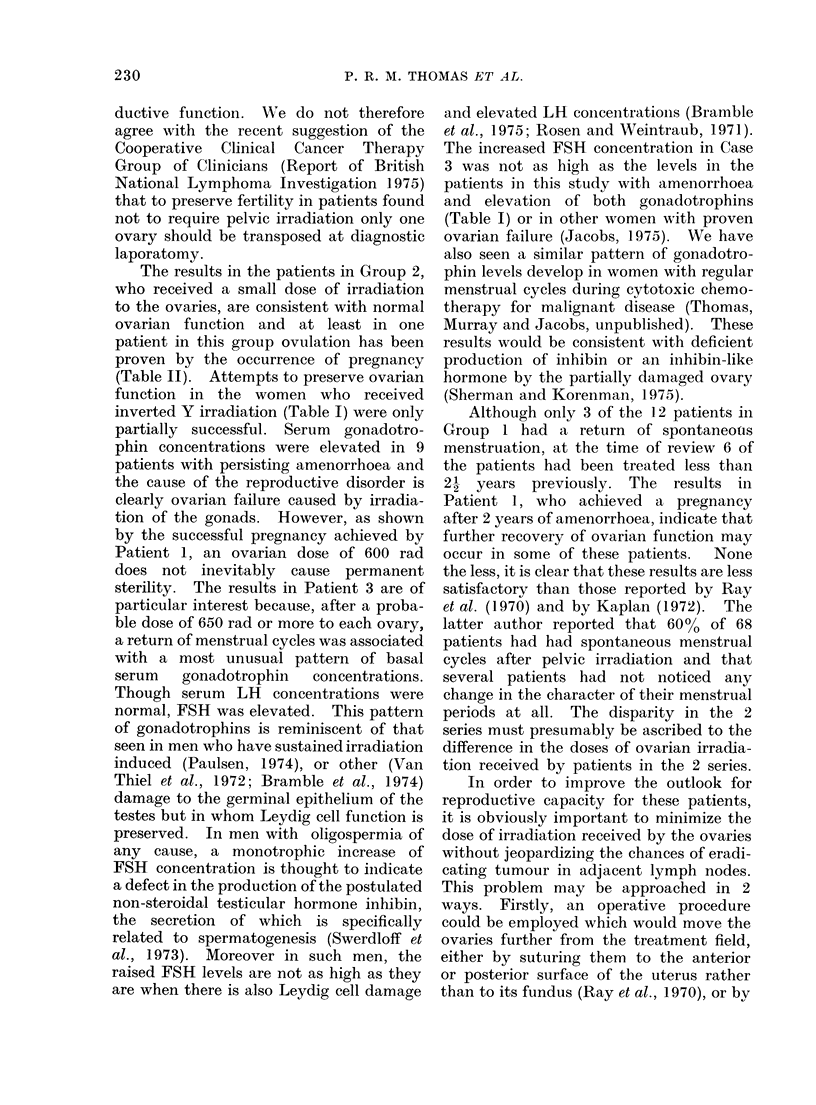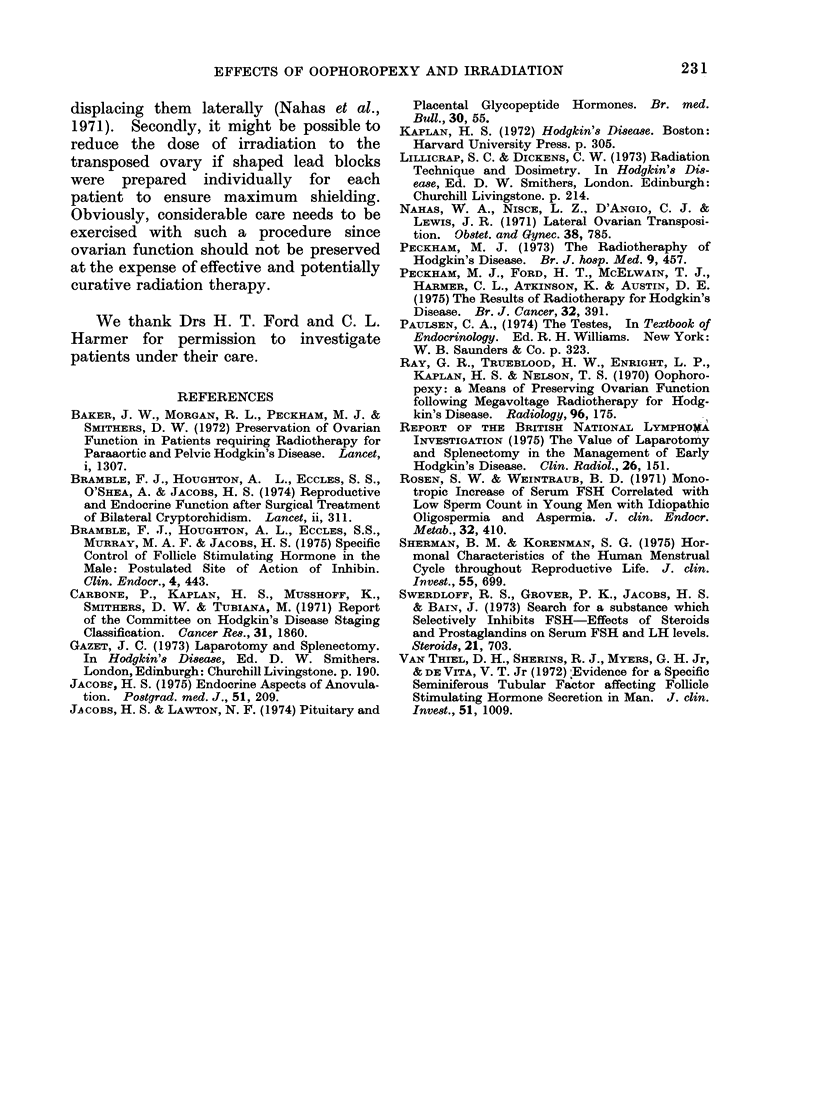# Reproductive and endocrine function in patients with Hodgkin's disease: effects of oophoropexy and irradiation.

**DOI:** 10.1038/bjc.1976.29

**Published:** 1976-02

**Authors:** P. R. Thomas, D. Winstanly, M. J. Peckham, D. E. Austin, M. A. Murray, H. S. Jacobs

## Abstract

Reproductive and endocrine function was investigated in 22 women with Hodgkin's disease who had bilateral mid-line oophoropexies performed at staging laparotomy. The operation was followed in 12 cases by "inverted Y" pelvic lymph node irradiation and in 4 cases by para-aortic lymph node irradiation. Pregnancies occurred after the operation in 4 of the 6 patients subsequently found not to require irradiation below the diaphragm. In the other 2 patients in this group the menstrual history was unaffected and normal gonadotrophin concentrations indicated intact ovarian function. In the group receiving para-aortic irradiation, in whom the ovarian irradiation dose was was small (about 150 rad to each ovary) menstrual function and gonadotrophin concentrations were normal at the time of review and one patient has subsequently become pregnant. In the group receiving inverted Y irradiation, in whom the ovaries were shielded from the radiation beam by a rectangular lead block, the ovarian dose was much higher (lowest dose 600 rad, highest dose 3500 rad). Nine of the 12 have persisting amenorrhoea with elevated levels of both gonadotrophins. One patient has since become pregnant and one patient has resumed menstrual cycles and has normal basal gonadotrophin concentrations. One patient who has resumed menstrual cycles has a monotrophic elevation of basal serum FSH concentrations. We conclude that bilateral mid-line oophoropexy does not impair ovarian function or gamete transport and should be performed at diagnositc laparotomy in women of child bearing age with Hodgkin's disease, even when it is uncertain whether pelvic node irradiation will be necessary. The results in the patients who received inverted Y irradiation indicate that the technique of pelvic shielding and ovarian transposition used were only partially successful in preserving fertility. Alternative techniques for preserving ovarian function are discussed.


*Br. J. Cancer* (1976) **33**, 226

### REPRODUCTIVE AND ENDOCRINE FUNCTION IN PATIENTS WITH HODGKIN'S DISEASE: EFFECTS OF OOPHOROPEXY AND IRRADIATION

P. R. M. THOMAS, D. WINSTANLY, M. J. PECKHAM, D. E. AUSTIN, M. A. F. MURRAY* AND H. S. JACOBS*

*From the Institute of Cancer Research and Royal Mardsen Hospital, London and Surrey, and the *Department of Obstetrics and Gynaecology, St Mary's Hospital Medical School, London W2*

Received 29 September 1975 Accepted 15 October 1975

**Summary.—Reproductive and endocrine function was investigated in 22 women with Hodgkin's disease who had bilateral mid-line oophoropexies performed at staging laparotomy. The operation was followed in 12 cases by " inverted Y " pelvic lymph node irradiation and in 4 cases by para-aortic lymph node irradiation.**

**Pregnancies occurred after the operation in 4 of the 6 patients subsequently found not to require irradiation below the diaphragm. In the other 2 patients in this group the menstrual history was unaffected and normal gonadotrophin concentrations indicated intact ovarian function. In the group receiving para-aortic irradiation, in whom the ovarian irradiation dose was small (about 150 rad to each ovary) menstrual function and gonadotrophin concentrations were normal at the time of review and one patient has subsequently become pregnant. In the group receiving inverted Y irradiation, in whom the ovaries were shielded from the radiation beam by a rectangular lead block, the ovarian dose was much higher (lowest dose 600 rad, highest dose 3500 rad). Nine of the 12 have persisting amenorrhoea with elevated levels of both gonadotrophins. One patient has since become pregnant and one patient has resumed menstrual cycles and has normal basal gonadotrophin concentrations. One patient who has resumed menstrual cycles has a monotrophic elevation of basal serum FSH concentrations.**

**We conclude that bilateral mid-line oophoropexy does not impair ovarian function or gamete transport and should be performed at diagnostic laparotomy in women of child bearing age with Hodgkin's disease, even when it is uncertain whether pelvic node irradiation will be necessary. The results in the patients who received inverted Y irradiation indicate that the technique of pelvic shielding and ovarian transposition used were only partially successful in preserving fertility. Alternative techniques for preserving ovarian function are discussed.**

MODERN methods of treatment of Hodgkin's disease have greatly improved the prognosis even in patients with advanced disease. The results of treatment by radiotherapy for pathologically staged (PS) nodal disease (Carbone *et al.*, 1971) at the Royal Marsden Hospital show that approximately 80% of patients remain disease free, although more time is needed to assess the long-term prospects for these patients (Peckham *et al.*, 1975). Preliminary results for PS IIIA cases are similar to those for PS I and II. Patients with abdominal node and/or splenic involvement receive irradiation to the lymph nodes along the iliac vessels and, since the ovaries lie in close proximity to this area, if measures are not taken to ensure they are moved away from the radiation beam they receive a dose of the order 3500 rad. This dose inevitably causes ovarian failure.

In an attempt to preserve ovarian function in young women receiving pelvic node irradiation, we have employed a technique of mid-line ovarian transposition (oophoropexy). The subsequent menstrual history of women who have had this procedure followed by pelvic node irradiation has been described in publications from the Stanford University Medical Center (Ray *et al.*, 1970; Kaplan, 1972) and in a previous report from the Royal Marsden Hospital (Baker *et al.*, 1972). The purpose of the present study was to compare reproductive and endocrine function in patients having bilateral oophoropexies with those having this operation followed by infradiaphragmatic irradiation.

### PATIENTS AND METHODS

The records of 33 patients having oophoropexies at the Royal Marsden Hospital between 1965 and 1974 were examined. Eleven cases were excluded from further study because of death, treatment with cytotoxic drugs (either alone or in addition to irradiation), or because they were taking oral contraceptives at the time of review. The findings in the remaining 22 patients with Hodgkin's disease and in one patient with a non-Hodgkin's lymphoma (Patient 6, who was treated as if she had had Hodgkin's disease) are the subjects of this report.

In all patients both ovaries with their vascular pedicles were sutured to the fundus of the uterus (Gazet, 1973). The transposed ovaries were marked with silver clips placed laterally on the edge of each ovary so that their positions were visible on the treatment film. In 3 patients the procedure was performed electively, but since 1970 it has been carried out routinely at staging laparotomy in women of child-bearing age at the Royal Marsden Hospital.

*Radiotherapy*.—The patients were divided into 3 groups. Group 1 consisted of 12 patients with histological evidence of disease below the diaphragm who were treated with " inverted Y " radiotherapy (Peckham, 1973). A mid-plane dose of 3500 rad was delivered to the para-aortic, iliac and inguinal nodes. A mid-line pelvic shield consisting of an 8 cm thick rectangular lead block was used in every patient. Its dimensions depended upon the size of the pelvis and the proximity of adjacent lymph nodes (Fig. 1).

Group 2 consisted of 4 patients who had no histological evidence of disease below the diaphragm but who received elective para-aortic irradiation for reasons described elsewhere (Peckham *et al.*, 1975). The patients

FIG. 1.—The relationship of the inverted Y irradiation field to the clips denoting the transposed ovaries.

in both groups were treated with an 8 MeV linear accelerator using parallel opposed anterior and posterior fields. The inferior limit of the para-aortic field was the 5th lumbar vertebra (Fig. 2). A dose of 3500 rad was delivered and no attempt was made to shield the ovaries from external scattered irradiation.

Group 3 consisted of 6 patients who had bilateral oophoropexies but who received no infradiaphragmatic irradiation.

Despite the sharp beam of the linear accelerator, some scattered irradiation is inveitable. Less than 2% of the delivered dose is transmitted through the lead blocks and the major proportion of the ovarian dose arises from internal scatter from adjacent treated tissues (Lillicrap and Dickens, 1973). An estimate of the maximum dose received by the transposed ovaries was made by measurements in a phantom and by noting the dimensions of the shield and the position of the marker clips. The doses received by the transposed ovaries were calculated to the nearest 50 rad.

*Assessment of reproduction and endocrine function*.—Reproductive function after treatment was considered normal in those patients who subsequently became pregnant. The

FIG. 2.—The relationship of the para-aortic lymph node irradiation field to the clips denoting the transposed ovaries.

menstrual history was recorded and, in those who did not become pregnant, basal serum gonadotrophin concentrations were measured. Serum concentrations of luteinizing hormone (LH) and follicle stimulating hormone (FSH) were measured by radioimmunoassay (Jacobs and Lawton, 1974) using specific antisera kindly supplied by Dr W. D. Odell, and reference preparations provided by the Medical Research Council of the United Kingdom. In our laboratory during the follicular phase, normal serum FSH (MRC 68/39) is 25 ± 12 ng/ml (s.d.) and normal serum LH (MRC 68/40) is 1·5 ± 0·25 ng/ml (s.d.) In patients with proven ovarian failure the concentrations exceed 200 ng/ml and 6 ng/ml respectively.

RESULTS

Table I shows the reproductive and endocrine data in the 12 patients in Group 1. At the time of writing 9 have amenorrhoea associated with elevated levels of LH and FSH. One patient (Case 1) had a successful pregnancy following a 2-year period of amenorrhoea and 2 others (Patients 3 and 6) resumed spontaneous menstrual cycles after 2 years and after 6 months of amenorrhea respectively. Case 3 has an unusual pattern of basal serum gonadotrophin concentrations with a normal serum LH level but an FSH concentration that is raised. However, the FSH concentration is not as high as is usually seen in patients with ovarian failure (Jacobs, 1975) or as seen in other patients in this series with elevations of both gonadotrophins. The lowest maximum dose of irradiation sustained by individual ovaries of the patients in this group was 500 rad.

TABLE I.—*Reproductive and Endocrine Function following Oophoropexy and " Inverted Y " Irradiation*

| Case No. | Age and year of operation | | Estimated dose to ovary (rad) Left | Right | Menstrual history following operation | Serum LH (ng/ml) | Serum FSH (ng/ml) |
|---|---|---|---|---|---|---|---|
| 1 | 24 | 1965 | 650 | 700 | Amenorrhoea 2 years, then oligomenorrhoea 4 years, then pregnancy | * | * |
| 2 | 34 | 1967 | 650 | 650 | Amenorrhoea since irradiation | 11 | 620 |
| 3 | 32 | 1969 | No clips seen | | Amenorrhoea 2 years, then irregular menstruation | 1·1 | 160 |
| 4 | 36 | 1970 | 650 | Clip not seen | Amenorrhoea since irradiation | 6·0 | 700 |
| 5 | 18 | 1971 | 900 | 950 | Amenorrhoea since irradiation | 7·4 | 500 |
| 6 | 25 | 1971 | 600 | 600 | Amenorrhoea for 6 months, 1 year oral contraceptives then irregular menstruation | 0·7 | 37 |
| 7 | 32 | 1973 | 3500 | 3500 | Amenorrhoea since irradiation | 8·4 | 700 |
| 8 | 26 | 1973 | 3500 | 1250 | Amenorrhoea since irradiation | 7·2 | 500 |
| 9 | 20 | 1974 | 700 | 1000 | Amenorrhoea since irradiation | 11 | 390 |
| 10 | 31 | 1974 | 3500 | 3500 | Amenorrhoea since irradiation | 9·0 | 360 |
| 11 | 29 | 1974 | 800 | Clip not seen | Amenorrhoea since irradiation | 4·2 | 390 |
| 12 | 19 | 1974 | 1200 | 500 | Amenorrhoea since irradiation | 3·8 | 209 |

* Gonadotrophins not estimated. Normal reproductive function proved by pregnancy.

TABLE II.—*Reproductive and Endocrine Function following Oophoropexy and Para-aortic Lymph Node Irradiation*

| Case No. | Age and year of operation | | Estimated dose to ovaries Left | Right | Menstrual history following operation | Serum LH (ng/ml) | Serum FSH (ng/ml) |
|---|---|---|---|---|---|---|---|
| 13 | 20 | 1972 | 150 | Clip not seen | Normal menstrual cycles throughout | 0·7 | 16 |
| 14 | 29 | 1973 | No clips seen | | Period lighter for 6 months then normal | 0·6 | 29 |
| 15 | 31 | 1973 | Clip not seen | 150 | Initially irregular periods, then menorrhagia 6 months, now normal.* | 1·0 | 28 |
| 16 | 23 | 1974 | Clip not seen | 150 | Irregular menstruation for 3 months, then normal | 0·3 | 23 |

* This patient has become pregnant subsequent to these measurements

TABLE III.—*Reproductive and Endocrine Function following Oophoropexy in Those Patients who Received No Infradiaphragmatic Irradiation*

| No. | Age and year of operation | | Menstrual and reproductive history following operation | Serum LH ng/ml | Serum FSH ng/ml |
|---|---|---|---|---|---|
| 17 | 24 | (1970) | Normal periods throughout, no hormonal contraceptive used | 0·4 | 16 |
| 18 | 25 | (1970) | Pregnant in 1971, normal obstetric history | * | * |
| 19 | 26 | (1971) | Pregnant in 1974, normal obstetric history | * | * |
| 20 | 27 | (1972) | Pregnant in 1974, normal obstetric history | * | * |
| 21 | 28 | (1972) | Pregnant in 1974, normal obstetric history | * | * |
| 22 | 13 | (1973) | Normal periods throughout | 0·2 | 16 |

* Gonadotrophins not estimated. Normal reproductive function proven by pregnancy.

The results in Group 2 are shown in Table II. Though there were some initial menstrual disturbances in 3 patients, spontaneous menstrual cycles returned in all and serum gonadotrophin concentrations were normal at the time of review. One patient in this group subsequently became pregnant. The maximum estimated ovarian doses of irradiation in these patients were less than one-third of that of the patient with the lowest maximum ovarian dose in Group 1.

Four of the 6 patients having oophoropexies but no infradiaphragmatic irradiation have since had normal pregnancies (Table III.) One girl aged 13 has normal menstrual cycles and normal basal gonadotrophin concentrations, as does one patient (Case 17) who is avoiding conception with non-hormonal methods. All 5 children born to these patients since their operations and/or irradiation were normal at birth and have remained so up to the time of review.

### DISCUSSION

The results in Table III show that the technique of oophoropexy used at the Royal Marsden Hospital has itself no deleterious effect on reproductive function. Thus, 4 patients have had normal pregnancies since the operation, showing that the mechanical process of transposition has not affected transport of gametes. In the other 2 patients the normal basal gonadotrophins are an indication that ovarian function has been preserved. The policy of performing bilateral oophoropexies at staging laparotomy in these women with Hodgkin's disease of reproductive age, even if they are found subsequently not to require pelvic irradiation, is quite safe in terms of subsequent reproductive function. We do not therefore agree with the recent suggestion of the Cooperative Clinical Cancer Therapy Group of Clinicians (Report of British National Lymphoma Investigation 1975) that to preserve fertility in patients found not to require pelvic irradiation only one ovary should be transposed at diagnostic laporatomy.

The results in the patients in Group 2, who received a small dose of irradiation to the ovaries, are consistent with normal ovarian function and at least in one patient in this group ovulation has been proven by the occurrence of pregnancy (Table II). Attempts to preserve ovarian function in the women who received inverted Y irradiation (Table I) were only partially successful. Serum gonadotrophin concentrations were elevated in 9 patients with persisting amenorrhoea and the cause of the reproductive disorder is clearly ovarian failure caused by irradiation of the gonads. However, as shown by the successful pregnancy achieved by Patient 1, an ovarian dose of 600 rad does not inevitably cause permanent sterility. The results in Patient 3 are of particular interest because, after a probable dose of 650 rad or more to each ovary, a return of menstrual cycles was associated with a most unusual pattern of basal serum gonadotrophin concentrations. Though serum LH concentrations were normal, FSH was elevated. This pattern of gonadotrophins is reminiscent of that seen in men who have sustained irradiation induced (Paulsen, 1974), or other (Van Thiel *et al.*, 1972; Bramble *et al.*, 1974) damage to the germinal epithelium of the testes but in whom Leydig cell function is preserved. In men with oligospermia of any cause, a monotrophic increase of FSH concentration is thought to indicate a defect in the production of the postulated non-steroidal testicular hormone inhibin, the secretion of which is specifically related to spermatogenesis (Swerdloff *et al.*, 1973). Moreover in such men, the raised FSH levels are not as high as they are when there is also Leydig cell damage and elevated LH concentrations (Bramble *et al.*, 1975; Rosen and Weintraub, 1971). The increased FSH concentration in Case 3 was not as high as the levels in the patients in this study with amenorrhoea and elevation of both gonadotrophins (Table I) or in other women with proven ovarian failure (Jacobs, 1975). We have also seen a similar pattern of gonadotrophin levels develop in women with regular menstrual cycles during cytotoxic chemotherapy for malignant disease (Thomas, Murray and Jacobs, unpublished). These results would be consistent with deficient production of inhibin or an inhibin-like hormone by the partially damaged ovary (Sherman and Korenman, 1975).

Although only 3 of the 12 patients in Group 1 had a return of spontaneous menstruation, at the time of review 6 of the patients had been treated less than $2\frac{1}{2}$ years previously. The results in Patient 1, who achieved a pregnancy after 2 years of amenorrhoea, indicate that further recovery of ovarian function may occur in some of these patients. None the less, it is clear that these results are less satisfactory than those reported by Ray *et al.* (1970) and by Kaplan (1972). The latter author reported that 60% of 68 patients had had spontaneous menstrual cycles after pelvic irradiation and that several patients had not noticed any change in the character of their menstrual periods at all. The disparity in the 2 series must presumably be ascribed to the difference in the doses of ovarian irradiation received by patients in the 2 series.

In order to improve the outlook for reproductive capacity for these patients, it is obviously important to minimize the dose of irradiation received by the ovaries without jeopardizing the chances of eradicating tumour in adjacent lymph nodes. This problem may be approached in 2 ways. Firstly, an operative procedure could be employed which would move the ovaries further from the treatment field, either by suturing them to the anterior or posterior surface of the uterus rather than to its fundus (Ray *et al.*, 1970), or by displacing them laterally (Nahas *et al.*, 1971). Secondly, it might be possible to reduce the dose of irradiation to the transposed ovary if shaped lead blocks were prepared individually for each patient to ensure maximum shielding. Obviously, considerable care needs to be exercised with such a procedure since ovarian function should not be preserved at the expense of effective and potentially curative radiation therapy.

We thank Drs H. T. Ford and C. L. Harmer for permission to investigate patients under their care.

### REFERENCES

BAKER, J. W., MORGAN, R. L., PECKHAM, M. J. & SMITHERS, D. W. (1972) Preservation of Ovarian Function in Patients requiring Radiotherapy for Paraaortic and Pelvic Hodgkin's Disease. *Lancet*, i, 1307.

BRAMBLE, F. J., HOUGHTON, A. L., ECCLES, S. S., O'SHEA, A. & JACOBS, H. S. (1974) Reproductive and Endocrine Function after Surgical Treatment of Bilateral Cryptorchidism. *Lancet*, ii, 311.

BRAMBLE, F. J., HOUGHTON, A. L., ECCLES, S.S., MURRAY, M. A. F. & JACOBS, H. S. (1975) Specific Control of Follicle Stimulating Hormone in the Male: Postulated Site of Action of Inhibin. *Clin. Endocr.*, **4**, 443.

CARBONE, P., KAPLAN, H. S., MUSSHOFF, K., SMITHERS, D. W. & TUBIANA, M. (1971) Report of the Committee on Hodgkin's Disease Staging Classification. *Cancer Res.*, **31**, 1860.

GAZET, J. C. (1973) Laparotomy and Splenectomy. In *Hodgkin's Disease*, Ed. D. W. Smithers. London, Edinburgh: Churchill Livingstone. p. 190.

JACOBS, H. S. (1975) Endocrine Aspects of Anovulation. *Postgrad. med. J.*, **51**, 209.

JACOBS, H. S. & LAWTON, N. F. (1974) Pituitary and Placental Glycopeptide Hormones. *Br. med. Bull.*, **30**, 55.

KAPLAN, H. S. (1972) *Hodgkin's Disease*. Boston: Harvard University Press. p. 305.

LILLICRAP, S. C. & DICKENS, C. W. (1973) Radiation Technique and Dosimetry. In *Hodgkin's Disease*, Ed. D. W. Smithers, London. Edinburgh: Churchill Livingstone. p. 214.

NAHAS, W. A., NISCE, L. Z., D'ANGIO, C. J. & LEWIS, J. R. (1971) Lateral Ovarian Transposition. *Obstet. and Gynec.* **38**, 785.

PECKHAM, M. J. (1973) The Radiotheraphy of Hodgkin's Disease. *Br. J. hosp. Med.* **9**, 457.

PECKHAM, M. J., FORD, H. T., MCELWAIN, T. J., HARMER, C. L., ATKINSON, K. & AUSTIN, D. E. (1975) The Results of Radiotherapy for Hodgkin's Disease. *Br. J. Cancer*, **32**, 391.

PAULSEN, C. A., (1974) The Testes, In *Textbook of Endocrinology*. Ed. R. H. Williams. New York: W. B. Saunders & Co. p. 323.

RAY, G. R., TRUEBLOOD, H. W., ENRIGHT, L. P., KAPLAN, H. S. & NELSON, T. S. (1970) Oophoropexy: a Means of Preserving Ovarian Function following Megavoltage Radiotherapy for Hodgkin's Disease. *Radiology*, **96**, 175.

REPORT OF THE BRITISH NATIONAL LYMPHOMA INVESTIGATION (1975) The Value of Laparotomy and Splenectomy in the Management of Early Hodgkin's Disease. *Clin. Radiol.*, **26**, 151.

ROSEN, S. W. & WEINTRAUB, B. D. (1971) Monotropic Increase of Serum FSH Correlated with Low Sperm Count in Young Men with Idiopathic Oligospermia and Aspermia. *J. clin. Endocr. Metab.*, **32**, 410.

SHERMAN, B. M. & KORENMAN, S. G. (1975) Hormonal Characteristics of the Human Menstrual Cycle throughout Reproductive Life. *J. clin. Invest.*, **55**, 699.

SWERDLOFF, R. S., GROVER, P. K., JACOBS, H. S. & BAIN, J. (1973) Search for a substance which Selectively Inhibits FSH—Effects of Steroids and Prostaglandins on Serum FSH and LH levels. *Steroids*, **21**, 703.

VAN THIEL, D. H., SHERINS, R. J., MYERS, G. H. Jr, & DE VITA, V. T. Jr (1972) Evidence for a Specific Seminiferous Tubular Factor affecting Follicle Stimulating Hormone Secretion in Man. *J. clin. Invest.*, **51**, 1009.